# A chromosome-level reference genome of the Antarctic blackfin icefish *Chaenocephalus aceratus*

**DOI:** 10.1038/s41597-023-02561-w

**Published:** 2023-09-26

**Authors:** Seung Jae Lee, Jinmu Kim, Eun Kyung Choi, Euna Jo, Minjoo Cho, Jeong-Hoon Kim, Hyun Park

**Affiliations:** 1 Department of Biotechnology, College of Life Sciences and Biotechnology, Korea University, Seoul, Korea; 2 Korea Polar Research Institute (KOPRI), Yeonsu-gu, Incheon, Korea

**Keywords:** Next-generation sequencing, Eukaryote

## Abstract

The blackfin Icefish (*Chaenocephalus aceratus*) belongs to the family Channichthyidae and the suborder Notothenioidei which lives in the Antarctic. We corrected the mis-scaffolds in the previous linkage map results by Hi-C analysis to obtain improved results for chromosome-level genome assembly. The final assembly analysis resulted in a total of 3,135 scaffolds, a genome size of 1,065.72 Mb, and an N50 of 33.46 Mb. 820.24 Mb, representing 88.88% of the total genome, is anchored to 24 chromosomes. The final gene set of 38,024 genes, including AFGPs, was annotated using RNA evidence, proteins, and ab-initio predictions. The complete percentage of BUSCO analysis is 92.7%. In this study, we aim to contribute to the study of polar fishes by improving the genome sequences of the blackfin icefish with the AFGP genes belonging to the Notothenoidei.

## Background & Summary

The Antarctic Ocean is a very cold and difficult place for any species to survive. The seawater temperature is at subzero levels even in summer, and the intertidal ecosystem does not function because ice covers the shoreline and coastal waters to depths ≥30 m. However, some species can survive in these extreme environments. The Antarctic marine fish fauna consists of approximately 275 species, 95 of which belong to the perciform suborder Notothenioidei. Some species have unusual adaptations, such as the presence of antifreeze glycoprotein (AFGP) in their blood or the absence of hemoglobin, to survive under these frigid conditions^1,2^. The blackfin icefish is a species of crocodile icefish belonging to the family Channichthyidae and the suborder Notothenioidei. Its natural habitat ranges from Southern Georgia to the northern part of the Antarctic Peninsula in the Atlantic sector of the Southern Ocean and Bouvetøya Island. It is found in shelf waters to a depth of 450–770 m^3^. Blackfin icefish species have thin, highly vascularized, scaleless skin; elongated bodies; and a weaker skeleton in comparison with most red-blooded notothenioid species. Their body structure makes them extremely vulnerable to injury^4^. Icefish, also known as white-blooded fish, belong to a unique family in that they are the only known vertebrates to lack hemoglobin. Consequently, their blood oxygen-carrying capacity is just 10% of that of other teleosts. The blood of the blackfin icefish *Chaenocephalus aceratus* has significantly fewer erythrocytes. The blood sample of *C. aceratus* does not have a trace of red color. Instead, it has a translucent whitish color. The plasma is clear. The cell mass at the bottom of a centrifuged hematocrit tube has been reported to be creamy white, accounting for approximately 1% of the blood content^5^. The 15 known species of the notothenioid family Channichthyidae, including *C. aceratus*, have the same diploid number of chromosomes (2n = 48), predominantly acrocentric chromosomes^6^.

A previous study^7^ reported the genome assembly of the blackfin icefish and published its genetic linkage map. However, its chromosome-level genome assembly remains unknown. Here, we report the upgraded chromosome-level whole-genome assembly of the blackfin icefish using the Hi-C approach with the tissue of the same individual used in the previous study. The genome assembly was highly consistent with the genetic linkage map at the chromosome level, and some mis-scaffolding in the genetic linkage map was rectified. We compared the chromosome-level genome sequence with that of another icefish, the South Georgia icefish (*Pseudochaenichthys georgianus*), to verify chromosomal conformity. For assessing chromosomal stability, we compared the sequences with those of medaka (*Oryzias latipes*), torafugu (*Takifugu rubripes*), and stickleback (*Gasterosteus aculeatus*). Moreover, to perform gene prediction more accurately, we reconstructed the annotation process using the integrated process of GeneMark^8^ and PASA pipeline^9^ with EVidenceModeler^10^. Using the customized prediction process, we predicted the functions of 10 copies of trypsinogen genes, nine copies of antifreeze glycoprotein (AFGP) genes, and two copies of AFGP/trypsinogen-like protease chimeric genes, and a trypsinogen-like protease gene with high tandem duplication at intron and exon levels.

## Methods

### Hi-C sequencing

Tissue sample of blackfin Icefish from the same individuals used in the previous study^7^ were used for Hi-C analysis. The Dovetail^TM^ Hi-C library was prepared using the Dovetail^TM^ Hi-C Library Kit (Dovetail Genomics, Santa Crus, CA, USA), according to the manufacturer’s instructions. Ground tissue (250 mg) was crosslinked with PBS/formaldehyde; the chromatin sample was then prepared with SDS and wash buffer. After normalizing the chromatin sample, 800 ng of chromatin was used to prepare the library. The chromatin was picked up using chromatin capture beads and then digested using a restriction enzyme. The end was labeled with biotin and ligated to form intra-aggregated DNA. After cross-link reversal, 200 ng of DNA was sheared using the Covaris system (Covaris Inc., Woburn, MA, USA). Sheared DNA fragments were end-repaired and ligated using an Illumina adapter. Ligated DNA was purified using streptavidin magnetic beads. Purified DNA was then amplified via PCR to enrich the fragments. Capillary electrophoresis verified the amplified libraries’ quality (Bioanalyzer System, Agilent Technologies, Palo Alto, CA, USA). Sequencing was performed using the Illumina NovaSeq 6000 system (Illumina Inc., San Diego, CA, USA), according to the protocols provided for 2 × 150 sequencing^11^.

### Hi-C analysis with previous draft assembly

**Fig. 1:**
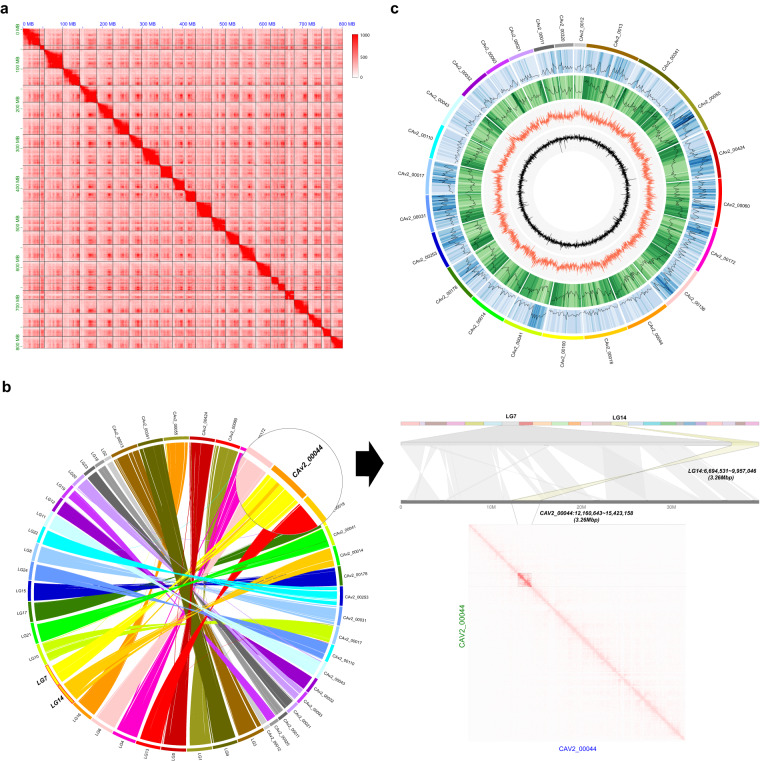
Summary of the final genome assembly results. (**a**) Contact map plot of the blackfin icefish genome. The Hi-C raw read pairs were aligned with the genome sequences. The x and y axes indicate their positions. The red dots indicate the position of the read pairs, and a high density of red dots denotes that they are located on the same chromosome. (**b**) Correction of mis-scaffolding of the linkage group in the blackfin icefish genome by Hi-C analysis. Mis-scaffolding of the LG14 linkage group was confirmed by Hi-C analysis. The 3.26M-sized sequence of LG14 was located on part of LG7, and the high density of linkage (red dot) was confirmed on the contact map at the position. (**c**) Overview of the blackfin icefish genome. The features are arranged in the order of gene density, repeat density, GC contents, and GC skew from outside to inside at 1-Mb intervals across the 24 chromosomes.

HiRise software, a pipeline for performing scaffolding analysis using proximity ligation data produced using the draft genome assembly, and Dovetail Hi-C technology were used for chromosome-level genome assembly^12^. The Hi-C reads were aligned to the draft assembly using SNAP. The positions of the mapped read pairs were used to construct a likelihood model of the genomic distance between read pairs. Genomic linking information between contigs was generated using the model and misjoins were corrected to construct a pseudomolecule-level scaffold genome. Juicer v.1.5.7^13,14^ was used to generate a hic file containing contact matrices with duplicate removal from the linking data. The Hi-C raw sequence data were aligned using BWA-MEM^15^. A contact map plot was drawn in detail using Juicebox v.1.5^13^, with the Juicer output being a hic file. Dovetail^TM^ HiRise allowed the upgrade from draft genome assembly to chromosome-level genome assembly within 24 chromosomal sequences (Fig. 1a). The longest scaffold length was 48 Mb, and the scaffold N50 value was 33 Mb (Table 1). We confirmed that there were 24 scaffolds of ≥10 Mb, consistent with the number of chromosomes in the blackfin icefish (2n = 48). Moreover, the total size of unplaced scaffolds was 262.76 Mb (Table 2).

**Table 1:** Summary of the blackfin icefish genome assembly.

	Hi-C
Number of Scaffolds	3,135
Total Size of Scaffolds	1,065,717,810
Longest Scaffold Size	48,033,548
Number of Scaffolds >1 M nt	30
Number of Scaffolds >10 M nt	24
N50 Scaffold Length	33,456,537
L50 Scaffold Count	14
GC Contents (%)	42.08

**Table 2:** Summary of chromosome length of the blackfin icefish.

Chromosome	Scaffold ID	Length (bp)	Percentage of Length (%)
1	CAv2_00060	43,377,216	5.40%
2	CAv2_00012	10,071,477	1.25%
3	CAv2_00013	48,033,548	5.98%
4	CAv2_00172	42,161,075	5.25%
5	CAv2_00424	43,635,658	5.43%
6	CAv2_00136	39,914,057	4.97%
7	CAv2_00044	39,709,832	4.95%
8	CAv2_00031	33,022,785	4.11%
9	CAv2_00341	44,074,585	5.49%
10	CAv2_00017	31,552,415	3.93%
11	CAv2_00043	30,661,890	3.82%
12	CAv2_00032	28,277,769	3.52%
13	CAv2_00018	39,033,829	4.86%
14	CAv2_00014	34,506,046	4.30%
15	CAv2_00253	33,456,537	4.17%
16	CAv2_00055	43,775,710	5.45%
17	CAv2_00100	36,811,101	4.58%
18	CAv2_00011	18,079,535	2.25%
19	CAv2_00320	16,705,358	2.08%
20	CAv2_00093	22,951,855	2.86%
21	CAv2_00041	35,575,720	4.43%
22	CAv2_00178	33,538,320	4.18%
23	CAv2_00021	22,731,248	2.83%
24	CAv2_00110	31,303,070	3.90%
Total		802,960,636	100.00%
unplaced		262,757,174	32.72%

### Comparative genomics analysis

**Fig. 2:**
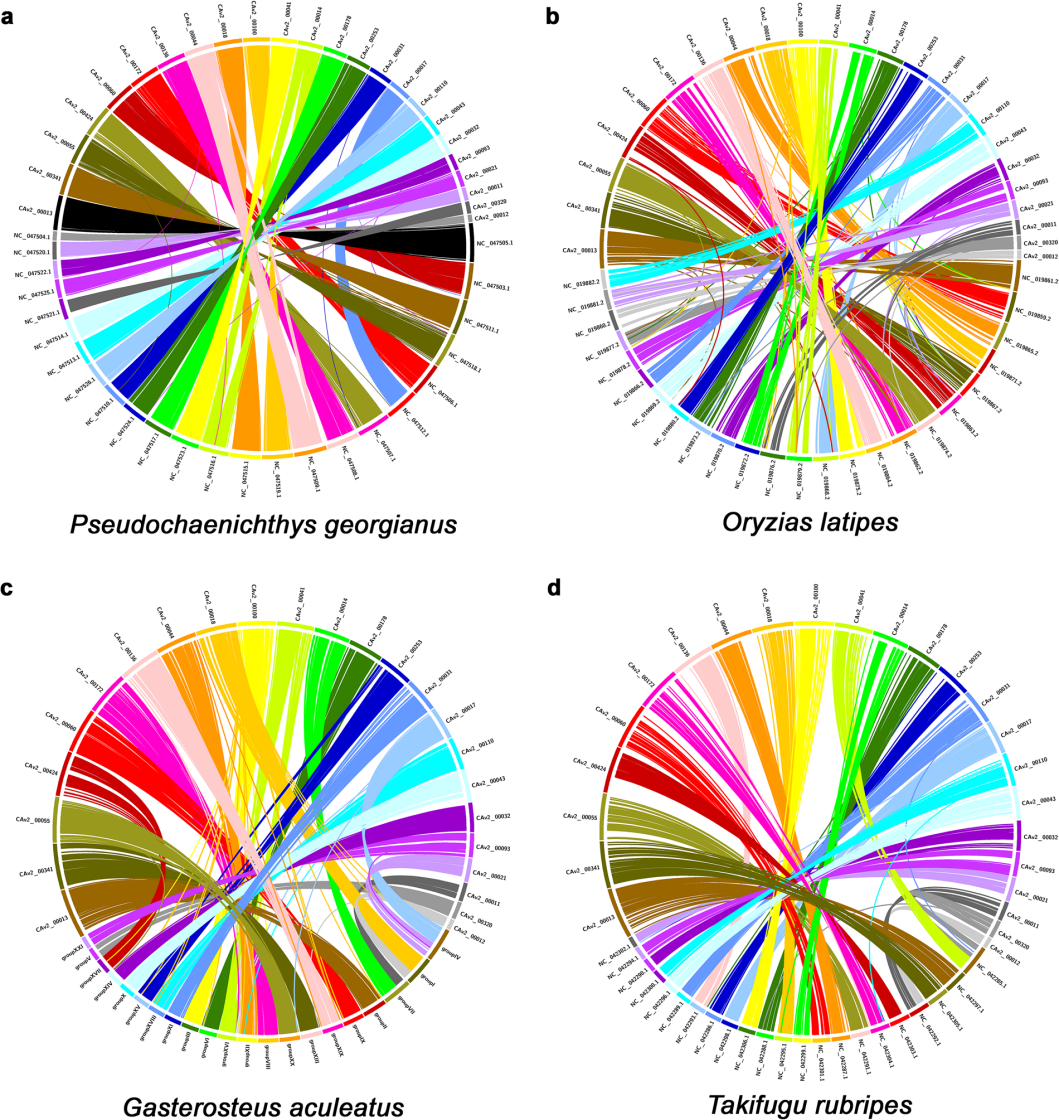
Chromosomal comparison with the blackfin icefish. P. georgianus (**a**) and O. latipes (**b**) which have the same number of chromosomes (2n = 48) were compared with the blackfin icefish. Chromosomal comparison of the blackfin icefish with G. aculeatus (**c**, 2n = 42) and T. rubripes (**d**, 2n = 44) which have less than the number of chromosomes.

To compare genome sequences at the chromosome level, nucmer in the MUMmer software package v.4.02b^16^ was used with the parameters -c 1000 -l 1000 and add--mum for unique matching and avoiding repeat regions. For a clear chromosome comparison, only long sequences corresponding to chromosomes were extracted and compared; unordered contig or scaffold sequences were excluded. Circos^17^ is a useful tool for comparing genome sequences based on homogeneous coordinates. In our study, a custom script was used to convert the coordinate data obtained through nucmer into a readable format in Circos. The results of chromosome comparison between two genomes were diagrammed using Circos. For visualizing detailed structural variation, GenomeRibbon^18^ was used to assess the coordinate data obtained through nucmer. To confirm the chromosomal stability of the Hi-C assembly, 24 chromosomes of the South Georgia icefish (*P. georgianus*)^19^ and medaka (*O. latipes*)^20^ genomes were compared with 24 chromosomes of the Hi-C assembly to assess their similarity. Each chromosome of the blackfin icefish was exclusively linked to each chromosome of the South Georgia icefish and medaka, thereby reconfirming the chromosomal stability of the scaffolds from the Hi-C assembly and verifying the integrity of the analysis (Fig. 2a,b). Antarctic fishes, including icefish species, diverged from the stickleback lineage approximately 77 million years ago^7^. For comparison with the chromosomes of the blackfin icefish, 21 stickleback chromosomes were aligned with the chromosome-level assembly. The results revealed that three chromosomes of the stickleback (*G. aculeatus*)^21^ were split into six chromosomes of the blackfin icefish (Fig. 2c). Moreover, 22 chromosomes of the pufferfish (*T. rubripes*)^22^ were compared with 24 chromosomes of the blackfin icefish. Pufferfish diverged from the Antarctic fish and stickleback lineages approximately 122 million years ago. Four chromosomes of the blackfin icefish (CAv2_00041, CAv2_00320, CAv2_00011, and CAv2_00012) were found to align with two chromosomes (chromosome 1:NC_042285.1 and chromosome 8: NC_042292.1) of pufferfish (Fig. 2d).

### Repeat analysis

A de novo repeat library was constructed using RepeatModeler v.1.0.3^23^, including RECON and RepeatScout v.1.0.5^24^, with default parameters. Moreover, Tandem Repeats Finder^25^ was used to predict consensus sequences and classification data for each repeat. All repeats collected by RepeatModeler were searched against the UniProt/SwissProt database^26^; transposons were excluded. To identify highly accurate long terminal repeat retrotransposons (LTR-RTs), an LTR library was constructed using LTR_retriever v.2.9.0^27^ with combined raw LTR data from LTRharvest^28^ with parameters ‘-minlenltr 100 -maxlenltr 7000 -mintsd 4 -maxtsd 6 -motif TGCA -motifmis 1 -similar 85 -vic 10 -seed 20’ and LTR_FINDER^29^ with parameters ‘-harvest_out -size 1000000 -time 300’. Repetitive elements were identified using RepeatMasker v.4.0.9 with a de novo repeat library with parameters ‘-no_is -norna’. Various TE subfamilies were detected in the genome, accounting for 52.88% of the analyzed genome. Their distribution was as follows: DNA transposons, 15.74%; long interspersed nuclear elements, 7.73%; short interspersed nuclear elements, 0.43%; LTRs, 16.92%; and unknown elements, 9.72% (Table 3).

**Table 3:** Summary of annotated transposable elements of the blackfin icefish.

Class	Count	Length occupied (bp)	Percentage of sequences
SINEs	29,839	4,538,018	0.43
LINEs	184,444	82,365,499	7.73
LTR elements	459,278	180,267,424	16.92
DNA elements	565,316	167,745,457	15.74
Unclassified	470,871	103,632,464	9.72
Small RNA	7,705	1,934,905	0.18
Satellites	9,349	1,838,578	0.17
Simple repeats	294,617	23,376,738	2.19
Low complexity	24,186	1,458,893	0.14
Total		563,522,826	52.88

### Gene prediction and annotation

Genome prediction was performed using EVidenceModeler (EVM) v.1.1.1^10^, which integrates the results of multiple gene predictions. Repeat masked genomes were used for ab initio gene prediction using GeneMark-ES v.4.68^30^ and Augustus v.3.4.0^31^. Then, the hints for protein and ab initio predictions were extracted with massive protein sequences from Actinopterygii, a clade of bony fishes, in the UniProt/SwissProt protein database^32^ using ProtHint v.2.6.0^8^. The hints were used to perform protein predictions using GeneMark-EP + v.4.68^8^ and ab initio predictions using Augustus. To obtain transcriptome-level evidence, the PASA pipeline v.2.3.3^9^ with Iso-Seq data was used. EVM was used to integrate the ab initio, transcriptome, and protein prediction results to obtain the final gene prediction with weight parameters ‘ABINITIO_PREDICTION = 1, PROTEIN = 50, TRANSCRIPT = 50’. Finally, to predict changes in exons by the addition of untranslated regions (UTRs), the PASA pipeline with Iso-Seq data was used again. Genome Annotation Generator v.2.0.1^33^ was used for adding start/stop codon data and generating a well-formed gff file. Other noncoding RNAs were identified using v.0.9. Putative tRNA genes were identified using tRNAscan-SE v.2.0.5^34^. The predicted genes were annotated by aligning them to the NCBI non-redundant protein (nr) database^35^ using NCBI BLAST v.2.9.0^36^ with a maximum e-value of 1e-5. To obtain protein domain information, InterProScan v.5.44.79^37^ was used with a protein sequence translated from a transcript. Moreover, Trinotate^38^ was used for the comprehensive annotation of transcriptome sequences, and TransDecoder v.5.5 with eggNOG (evolutionary genealogy of genes: Non-supervised Orthologous Groups) and KEGG (Kyoto Encyclopedia of Genes and Genomes) were used for decoded peptide sequences. Protein signal peptide prediction was performed using SignalP v.5.0^39^, and transmembrane domain prediction was performed using TMHMM v2.0^40^. Gene Ontology (GO) terms^26^ were assigned to the genes using the BLAST2GO pipeline v.4.0^41^. A total of 38,024 genes and 39,889 coding sequences (CDSs) were analyzed in the *C. aceratus* genome. The average length of CDSs was 1,248 bp, and the average number of exons per gene was 7.9 (Table 4). Consequently, a total of 39,889 CDSs were annotated from a minimum of 17.51% to a maximum of 90.31% in seven databases for functional annotation. In one or more databases, 79.03% of CDSs were annotated (Table 5). To confirm the gene prediction results, BUSCO was used in transcriptome mode with CDSs. The percentage of complete BUSCOs was 80.7%, while that of missing was 13.4% (Table 6).

**Table 4:** Summary of gene predictions of the blackfin icefish.

Features	Number of Features	Total Length of Features (bp)	Average Length of Features (bp)	Density (Features /Mbp)
Gene	38,024	389,488,764	10,243.2	35.683
CDS	39,889	49,787,931	1,248.2	37.434
Exon	299,280	59,564,228	199.0	280.857

**Table 5:** Summary of functional annotation of the blackfin icefish.

Database	Number of Annotations	Percent of Annotations
Uniprot/Swiss-prot	28,883	75.96
Gene Ontology	28,619	75.27
KEGG	25,598	67.32
Pfam	24,130	63.46
TmHMM	6,658	17.51
SignalP	34,340	90.31
EggNOG	24,831	65.30
1 > Databases	30,050	79.03

**Table 6:** Assessment of the blackfin icefish transcriptome and protein using BUSCO.

	Transcriptome		Protein	
	Number of BUSCOs	Percentage of BUSCOs	Number of BUSCOs	Percentage of BUSCOs
Complete BUSCOs	2,970	81.6	2926	80.4
Complete and single-copy BUSCOs	2,702	74.2	2658	73.0
Complete and duplicated BUSCOs	268	7.4	268	7.4
Fragmented BUSCOs	215	5.9	234	6.4
Missing BUSCOs	455	12.5	480	13.2

### Annotation of AFGP genes

**Fig. 3:**
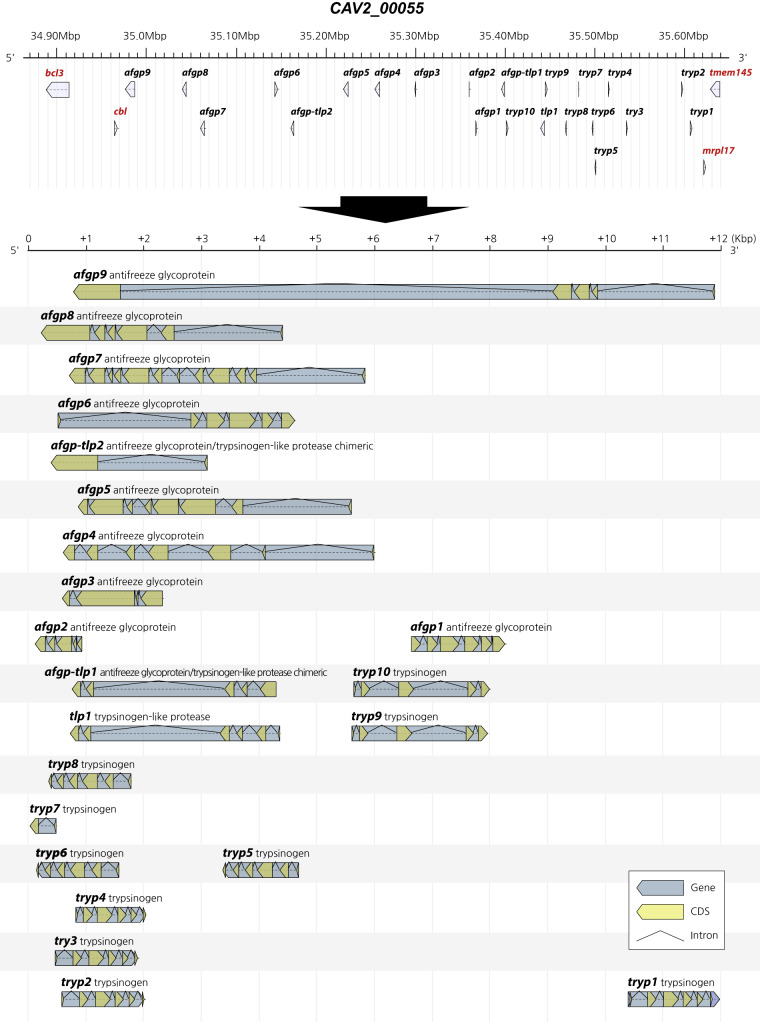
Antifreeze glycoprotein (AFGP) gene family for the blackfin icefish. AFGP gene family which has 22 genes was found on the blackfin icefish genome. It was identified in the region from 34,957,786 to 35,607,986 in the scaffold CAv2_00055 and contains 10 trypsinogen genes and 9 AFGP genes.

The regions containing AFGP and trypsinogen genes were extracted from the whole-genome sequence using NCBI BLAST v.2.9.0^36^ against transcript and protein sequences of the Antarctic toothfish^42^. AFGP genes were predicted using Exonerate v.2.4 with the following specific parameters:--model protein2genome--minintron 20--maxintron 10000--score 250--percent 60 from the extracted region sequence. The final AFGP gene set was identified based on identity, similarity, and alignment length and was integrated into the final gene prediction data. The sequence encoding AFGP, which is similar to the long repetition of simple sequences, is very repetitive and is not assembled in the short sequence of the next-generation sequence despite their high throughput sequences. We identified that genes encoding AFGP were tandemly duplicated in the Cav2_00055 scaffold from 34,915,108 bp to 35,620,009 bp. The AFGP–trypsinogen locus was located between genes encoding mitochondrial 39 S ribosomal protein L17 (mrpl17) and E3 ubiquitin-protein ligase CBL-C isoform X1 (cbl), as reported in a previous study. However, in this study, 10 copies of trypsinogen genes, nine copies of AFGP genes, two copies of AFGP/trypsinogen-like protease chimeric genes, and a trypsinogen-like protease gene were predicted at the exon/CDS level (Fig. 3). AFGP genes evolved from trypsinogen genes in Antarctic fishes^43^. The prediction of gene features of AFGP genes is too difficult by the normal automated prediction method because the AFGP gene sequence has a high incidence of tandem repeats. We developed a customized process to predict complete AFGP gene features and analyzed exons and CDSs of AFGP and trypsinogen genes. Our results were consistent with previous results, except in the case of one AFGP gene. Moreover, we obtained tandemly duplicated AFGP gene sequences. Using our developed method, further analysis of the AFGP genes of other Antarctic fishes can be performed.

## Data Records

The final genome assembly of the blackfin icefish was deposited at GeneBank (accession GCA_023974075.1)^44^. Also, the Hi-C raw data were deposited NCBI Sequence Read Archive (SRA) with accession number SRR24715329^11^.

## Technical Validation

We assessed the completeness of genome assembly using Benchmarking Universal Single-Copy Orthologs (BUSCO)^45^ v.5.4.4 with the Actinopterygii lineage dataset with default parameters. A total of 3,375 (92.7%) BUSCOs were identified as complete. Of these, 3,241 (89.0%) were single-copy and 134 (3.7%) were duplicated. The numbers of partially matched and missing were 48 (1.3%) and 217 (6.0%), respectively (Table 7). The k-mer completeness and quality value (QV) were evaluated by Merqury v1.3^46^. Merqury analysis were QV of 29.96 and completeness of 88.29 (Table 8). On comparing the Hi-C scaffolds and linkage groups, high concordance was noted; however, some inconsistencies remained. In particular, mis-scaffolding was noted between LG14 and LG17. Assessment of the Hi-C scaffold confirmed that the 3.26M-sized sequence located in LG14 (LG14: 6,694,531–9,957,046) was transferred to the middle of LG7 (CaV2_00044: 12,160,643–15,423,158). Moreover, the CaV2_00044 scaffold, which was consistent with LG7, was completely scaffolded on the Hi-C contact map (Fig. 1b). These results confirmed that the mis-scaffold on the linkage group was corrected through Hi-C analysis. Moreover, the Hi-C scaffold was verified with the contact map. Many linkage group-based genome assembly results have been improved or finalized for several years through Hi-C analysis^47,48^.

**Table 7:** Assessment of the blackfin icefish genome assembly using BUSCO.

	Number of BUSCOs	Percentage of BUSCOs
Complete BUSCOs	3,375	92.7
Complete and single-copy BUSCOs	3,241	89.0
Complete and duplicated BUSCOs	134	3.7
Fragmented BUSCOs	48	1.3
Missing BUSCOs	217	6.0

**Table 8:** Evaluation of the blackfin icefish genome using Merqury.

Quality Value (QV)	k-mer error rate	k-mer completeness (%)
29.9614	0.00100893	88.2895

## Data Availability

The bioinformatics analysis software used in this study was analyzed using the standard parameters provided by the software developers. If manually adjusted parameters were used, the software version and method used are described in the Methods.
