# Author Correction: A chromosome-level reference genome of the Antarctic blackfin icefish *Chaenocephalus aceratus*

**DOI:** 10.1038/s41597-023-02861-1

**Published:** 2024-01-08

**Authors:** Seung Jae Lee, Jinmu Kim, Eun Kyung Choi, Euna Jo, Minjoo Cho, Jeong-Hoon Kim, Hyun Park

**Affiliations:** 1 Department of Biotechnology, College of Life Sciences and Biotechnology, Korea University, Seoul, Korea; 2 Korea Polar Research Institute (KOPRI), Yeonsu-gu, Incheon, Korea

**Keywords:** Next-generation sequencing, Eukaryote

Correction to: *Scientific Data* 10.1038/s41597-023-02561-w, published online 26 September 2023

In this article the affiliation details for Hyun Park were incorrectly given as ‘Korea Polar Research Institute (KOPRI), Yeonsu-gu, Incheon, Korea’ but should have been ‘Department of Biotechnology, College of Life Sciences and Biotechnology, Korea University, Seoul, Korea’. The original article has been corrected.

